# SHARING THE CARE: SOCIAL MEDIA USE FOR SELF-DISCLOSURE IN FAMILY CAREGIVERS OF PEOPLE WITH DEMENTIA

**DOI:** 10.1093/geroni/igae098.3717

**Published:** 2024-12-31

**Authors:** Gianna Kohl, Katrina Scior, Georgina Charlesworth

**Affiliations:** University of Illinois Urbana-Champaign, Champaign, Illinois, United States; University College London, London, England, United Kingdom; University College London, London, England, United Kingdom

## Abstract

Family caregivers of people with dementia often face unique challenges in their caregiving role, including the complex task of sharing their caregiving experiences and identity with their social networks. Social media platforms provide a valuable avenue for self-disclosure of stigmatized identities such as dementia, enabling access to support. This study explores the patterns and purposes of self-disclosure by family caregivers of people with dementia on social media. A cross-sectional online survey was conducted with 323 respondents, aged 20 to 86 (M = 59.2, SD = 12.34), of whom 78% were female. Among the whole sample, 255 (79%) reported using social media, with 66% of them using it daily. Facebook was the most frequently used platform (81%), followed by Twitter (42%) and Instagram (33%). Of the social media users, 121 (47%) also posted dementia-related content. Content analysis of open-ended questions revealed that these caregivers use social media to share their dementia caregiving journey, including providing insights into the lives and experiences of the person they support, while also engaging in awareness raising and mutual support activities with other caregivers. The findings show that social media is an important resource for family caregivers of people with dementia, facilitating the sharing of personal experiences, building support networks, and engaging in advocacy. The high rate of regular use underscores its value in navigating caregiving experiences and challenges. Healthcare professionals and organizations should leverage these platforms to offer targeted support, while future research should explore how social media can be integrated into support strategies.

